# Evaluation on Emergency Medical Services Departure in the Trnava University Hospital, the Slovak Republic

**Published:** 2019-06

**Authors:** Jaroslav STANCIAK, Lubica VARECKOVA, Martin SAMOHYL

**Affiliations:** 1. Trnava University Hospital, Trnava, Slovak Republic; 2. Pedagogical Faculty, Comenius University, Bratislava, Slovak Republic; 3. Faculty of Social Sciences, University of Ss Cyril and Methodius, Trnava, Slovak Republic; 4. Institute of Hygiene, Faculty of Medicine, Comenius University, Bratislava, Slovak Republic

## Dear Editor-in-Chief

In compliance with the valid legislation of the Slovak Republic, the Emergency Medical Ambulance Services is divided into Emergency Health Services (EHS) managed by professional non-medical personnel, and Emergency Medical Services (EMS) managed by a specialized doctor. Several of the emergency medical services studies have been conducted (1–3).

This study analysed the average annual percent change (AAPC) of selected causes of ambulance departures (CAD) (%) in the period of eight years (2009–2016), divided into two sub-periods (2006–2009 and 2010–2016) at the *Department* of Pediatrics (The Trnava University Hospital).

The data were obtained from the Records on Person’s Health Assessment; where 1,831 CAD were analysed.

For the trend analysis, the AAPC indicator (4) was proposed. Thus, the mean percentage difference of annual growth/decline can be obtained, using the following equation:

$$\mathrm{AAPC}=\frac{1}{n}\sum \frac{x_{i}-x_{i-1}}{x_{i-1}}*100$$

where *x* represents input vector of CAD in period *n* in year *i*, in each analysed period.

For statistical evaluation of the systematic change significance in particular period, we used Theil-Sen estimator (5) of trend line based on median and its significance was tested by nonparametric Wilcoxon test using statistical software R package- Median-Based Linear Model *(mblm)*. The trend was considered significant if *P*-value was <0.05. The calculations and figures were made in statistical software R-project.

The AAPC of CAD trend at the *Department* of Pediatrics (The Trnava University Hospital), in particular periods, are given in Table 1. In the period of 2009–2012, the highest AAPC of CAD (22.3%) was observed due to collapses and unconsciousness (*P*<0.001), what was proved also in eight-year period (2019–2016), a permanent high significant increase of AAPC of CAD (*P*<0.01). The AAPC of CAD trend, trend due to exitus showed the most notable increase in the sub-period of 2013–2016 (22.2%; *P*<0.01). The AAPC of CAD trend due to diseases of urogenital tract showed the most notable decrease in the whole assessed period of 2009–2016 and the AAPC of CAD proved the significant negative values in sub-period 2009–2012 (−11.1%; *P*<0.01) and in sub-period 2003–2016 (−33.3%; *P*<0.001) and in females (−4.0%; P<0.001) (Table 1).

**Table 1:** The AAPC of CAD at the *Department* of Pediatrics (2009–2016)

***Selected causes of ambulance departure***	***2009*** ***n (%)***	***2010*** ***n (%)***	***2011*** ***n (%)***	***2012*** ***n (%)***	***2009–2012*** ***AAPC (%)***	***2013*** ***n (%)***	***2014*** ***n (%)***	***2015*** ***n (%)***	***2016*** ***n (%)***	***2013–2016*** ***AAPC (%)***	***2009–2016*** ***AAPC (%)***
Substances Intoxication	17 (9.6)	18 (8.4)	28 (12.4)	21 (10.1)	2,6	24 (10.2)	27 (11.0)	30 (12.0)	29 (10.5)	6.3*	5.4
Injuries	65 (36.7)	68 (31.8)	58 (25.7)	70 (33.8)	1,4	72 (30.5)	75 (30.6)	70 (28.0)	74 (26.8)	0.9	1.3
Respiratory diseases	22 (12.4)	27 (12.6)	37 (16.4)	29 (14.0)	6,0*	33 (14.0)	30 (12.2)	37 (14.8)	41 (14.9)	7.7*	7.0*
Cardiovascular diseases	5 (2.8)	2 (0.9)	3 (1.3)	0 (0.0)	-^a^	4 (1.7)	5 (2.0)	5 (2.0)	8 (2.9)	19.2**	-^a^
Collapses, unconsciousness	2 (1.1)	14 (6.5)	11 (4.9)	12 (5.8)	22.3***	11 (4.7)	14 (5.7)	15 (6.0)	18 (6.5)	15.1**	14.6**
Spasms	38 (21.5)	33 (15.4)	37 (16.4)	26 (12.6)	−15.5**	32 (13.6)	30 (12.2)	27 (10.8)	34 (12.3)	1.3	−3.6
Gastrointestinal diseases	12 (6.8)	16 (7.5)	15 (6.6)	15 (7.2)	6.1*	17 (7.2)	16 (6.5)	18 (7.2)	20 (7.2)	5.4*	6.4*
Mental diseases, neuro-circulatory asthenia	4 (2.3)	23 (10.7)	17 (7.5)	11 (5.3)	−2.4	19 (8.1)	24 (9.8)	22 (8.8)	23 (8.3)	5.6*	7.3*
Allergic reactions	4 (2.3)	5 (2.3)	5 (2.2)	6 (2.9)	12.2**	7 (3.0)	5 (2.0)	6 (2.4)	8 (2.9)	1.7	7.5*
Urogenital disease	0 (0.0)	3 (1.4)	1 (0.4)	3 (1.4)	−11.1**	4 (1.7)	2 (0.8)	3 (1.2)	2 (0.7)	−	-
										33.3***	17.9**
Other	7 (4.0)	5 (2.3)	13 (5.8)	14 (6.8)	9.6*	12 (5.1)	15 (6.1)	16 (6.4)	16 (5.8)	8.9*	5.5*
Exitus	1 (0.6)	0 (0.0)	1 (0.4)	0 (0.0)	-^a^	1 (0.4)	2 (0.8)	1 (0.4)	3 (1.1)	22.2**	-^a^
Total (n)	177	214	226	207	4.5*	236	245	250	276	5.0*	5.8*

* *P* < 0.05;

** *P* < 0.01;

*** *P* < 0.001

a impossible to evaluate

The authors believe that the present analysis of data from more than 1,831 CAD, and the use of methodology to evaluate AAPC rate could bring benefits to the Slovak epidemiological and public health literature regarding economic and social losses, and allows international comparisons of these national data.
